# Deep learning based analysis of G3BP1 protein expression to predict the prognosis of nasopharyngeal carcinoma

**DOI:** 10.1371/journal.pone.0315893

**Published:** 2025-01-27

**Authors:** Linshan Zhou, Mu Yang, Jiadi Luo, Hongjing Zang, Songqing Fan, Yuting Zhan

**Affiliations:** 1 Department of Nephrology, Hunan Key Laboratory of Kidney Disease and Blood Purification, The Second Xiangya Hospital of Central South University, Changsha, Hunan, China; 2 Anteeo Surgical, Beijing, China; 3 Department of Pathology, the Second Xiangya Hospital, Central South University, Changsha, Hunan, China; 4 Hunan Clinical Medical Research Center for Cancer Pathogenic Genes Testing and Diagnosis, Changsha, Hunan, China; Guangdong Medical University, CHINA

## Abstract

**Background:**

Ras-GTPase-activating protein (GAP)-binding protein 1 (G3BP1) emerges as a pivotal oncogenic gene across various malignancies, notably including nasopharyngeal carcinoma (NPC). The use of automated image analysis tools for immunohistochemical (IHC) staining of particular proteins is highly beneficial, as it could reduce the burden on pathologists. Interestingly, there have been no prior studies that have examined G3BP1 IHC staining using digital pathology.

**Methods:**

Whole-slide images (WSIs) were meticulously collected and annotated by experienced pathologists. A model was intricately designed and rigorously tested to yield the quantitative data regarding staining intensity and extent. The collective output data was subjected multiplicative analysis, exploring its correlation with the prognosis.

**Results:**

The G3BP1 molecular marker scoring model was successfully established utilizing deep learning methodologies, with a calculated threshold staining scores of 1.5. Notably, patients with NPC exhibiting higher expression levels of G3BP1 proteins displayed significantly lower for overall survival rates (OS). Multivariate analysis further validated that positive expression of G3BP1 stood as an independent poorer prognostic factors, indicating a poorer prognosis for NPC patients.

**Conclusion:**

Computational pathology emerges as a transformative tool capable of substantially reducing the burden on pathologists while concurrently enhancing and diagnostic sensitivity and specificity. The positive expression of G3BP1 protein serves as valuable, independent biomarker, offering predictive insights into a poor prognosis for patients with NPC.

## 1. Introduction

Nasopharyngeal carcinoma (NPC) stands as a unique form of head neck cancer, originating from the nasopharynx epithelium and exhibiting ethnic and geographical distribution preference [1, 2]. China’s cancer statistics for 2022 project an estimated 64,165 new cases and 36,315 cancer-related deaths attributable to NPC [3]. Our previous study corroborated the oncogenic role of the G3BP1, validating its promotion of proliferation, migration, and invasion both *in vitro* and *in vivo*. Clinical analysis further identified heightened G3BP1 expression as a robust prognostic marker, independently indicative of adverse outcomes for patients with NPC and non-small cell lung cancer (NSCLC) [4, 5]. Currently, immunohistochemical staining scoring relies heavily on semi-quantitative evaluation, typically conducted independently by two blinded researchers [6–8]. Nevertheless, this approach is prone to variations that result from pathologists’ experience and subjectivity. Furthermore, consistency and repeatability in the interpretation of evaluated markers across different laboratories are markedly disparate. Leveraging the advantages of computers in image processing, there exists the potential to enhance the accuracy and repeatability of the immunohistochemical scoring, thereby predicting patient prognosis and therapeutic response [9–11].

Deep learning approaches are gaining prominence as the cornerstone of machine-learning tools in medical imaging, demonstrating superior efficacy in tasks such as segmentation, classification, and prediction [12]. Notably, compared to traditional method, the deep learning image segmentation algorithms boast high accuracy and may mitigate insensitivity in the segmentation of gray histograms lacking evident double peaks [13]. Convolutional Neural Networks (CNNs), a subset of deep learning methods, have been proposed and optimized for image analysis, exhibiting state-of-the-art performance in various image classification tasks. In computational pathology. CNNs have demonstrated comparable proficiency to pathologists in image analysis tasks, including H&E-based PD-L1 prediction, H&E-based Her-2 prediction and IHC-based Her-2 scoring [10, 14, 15]. SOLOv2 is a simple, direct, fast and powerful instance segmentation model. SOLOv2 incorporates an efficient and comprehensive instance mask representation scheme, dynamically segmenting each image instance without relying on bounding box detection. Furthermore, it significantly enhances post-processing speed through a novel matrix non-maximum suppression technique [16, 17].

This study aims at constructing a reliable deep—learning framework for scoring G3BP1 and subsequently predicting prognosis. We developed and trained a G3BP1 status scoring model using 275 whole-slide images (WSI), divided into distinct sets for training, validation, and testing in a ratio of 6:2:2. Our pathology team meticulously annotated regions of interest (ROIs) within the WSIs. The output data encompassed staining intensity and staining extent, computed through computational pathology, demonstrating remarkable precision when compared to results manually labeled by pathologists. Importantly, our model exhibited exceptional proficiency in prognostic predictions for NPC patients. The high accuracy and reliability of the output data, coupled with the model’s ability to predict prognosis, underscore the potential of our deep learning framework in enhancing the understanding and management of G3BP1-related outcomes in NPC.

## 2. Materials and methods

### 2.1 Ethics statement

In this study, all samples were obtained with clear and explicit informed consent from the participants. Ethical oversight and approval for the study protocols, specimen utilization, and data retrieval were obtained from the Ethics Review Committee of the Second Xiangya Hospital of Central South University (Scientific and Research Ethics Committee, No. K022). We collected wax blocks and clinical data (They were stored in the Department of Pathology and computer information system of our hospital respectively) of the NPC patients under the guidance of the Ethics Committee after obtaining the Ethical oversight and approval. Written informed consent was secured from all adult patients, and for minors or children participating in the study, written informed consent was obtained from the next of kin, caretakers, or guardians. The study was conducted in accordance with established ethical guidelines, prioritizing participant confidentiality, and ensuring the responsible and ethical conduct of research throughout the investigation.

### 2.2 Patient cohorts and whole-slide images (WSI) acquisition

For this investigation, we randomly selected 275 cases of paraffin-embedded NPC, comprising 201 males and 74 females. These cases were drawn from the archives of the Department of Pathology at the Second Xiangya Hospital of Central South University (Changsha, China). All NPC specimens underwent rigorous pathological confirmation based on the World Health Organization’s histological classification of NPC. Importantly, none of patients had received prior radiotherapy or chemotherapy at the time of original biopsy. A comprehensive description of the patient cohort was showed in Table 1. WSI imaging was conducted using the Una digital pathology biopsy scanner, ensuring high-resolution and comprehensive representation of the tissue samples under examination. Such a standardized imaging contributes to the reliability and consistency of the dataset, crucial for the accuracy of subsequent deep learning analyses.

**Table 1:** Clinicopathological features of patients with NPC.

Patients features	No. of patients (%)
**NPC patients** **Gender**	
Male	201 (73.1)
Female	74 (26.9)
**Age**	
<40	56 (20.4)
≥40	219 (79.6 3)
**Clinical stages**	
I	3(1.1)
II	79 (28.7)
III	110 (40.0)
IV	83(30.2)
**LN status**	
No LNM	65 (23.6)
LNM(N1/N2/N3)	210 (76.4)
**Histological type**	
UDNPC	258 (93.8)
DNPC	17 (6.2)
**Survival status**	
Alive	238 (86.5)
Dead	37(13.5)

Abbreviations: DNPC: Differentiated non-keratinized nasopharyngeal carcinoma; UDNPC: Undifferentiated non-keratinized nasopharyngeal carcinoma; LN: lymph node; LNM, lymph node metastasis

### 2.3 Immunohistochemical staining

Immunohistochemical staining was performed using the HRP-Polymer anti-Mouse IHC Kit. Staining conditions for the antibody based on the expertise of our laboratory, as outlined in previous protocols [4, 7, 18]. This carefully selected antibody and dilution ratio were chosen to ensure optimal specificity and sensitivity in detecting G3BP1 expression, aligning with established practices in our laboratory. For this study, 1:300 dilution of primary antibody to G3BP1 (Monoclonal Mouse antibody, Catalog: sc-365338, SANTA CRUZ BIOTECHNOLOGY) were applied.

### 2.4 Model establishment

**Fig 1:**
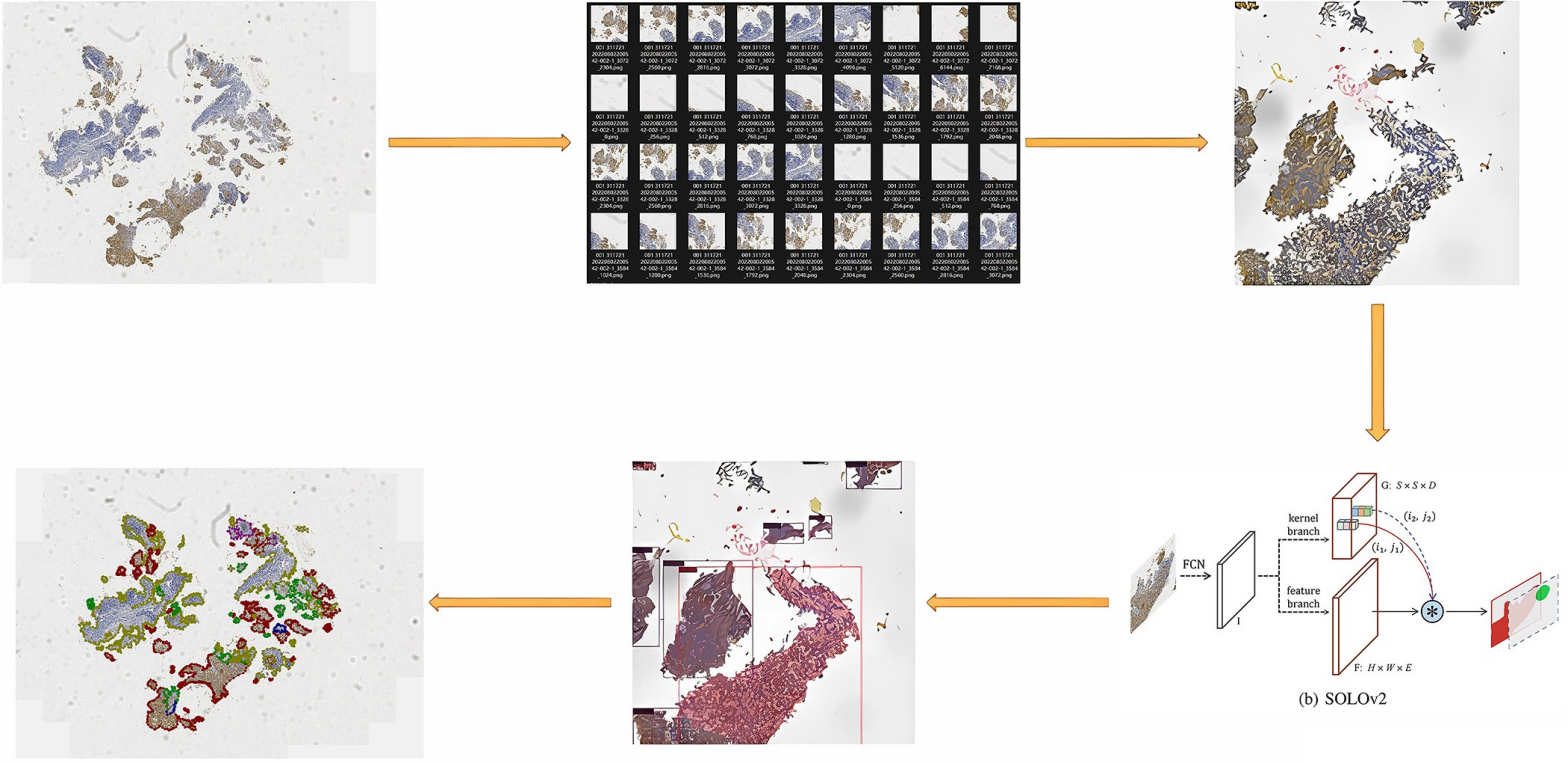
The entire process of the systematic steps involved in the model’s application. A full-film scan of the entire section was performed using a pathological biopsy scanner to generate Whole Slide Imaging (WSI). Each WSI image was segmented into patches of 1024*1024 pixels. Within each patch, it was performed segmentation and classification utilizing the SOLOv2 model. Subsequently, the outcomes were consolidated, and a detailed statistical analysis was carried out to quantify the total area and proportion occupied by distinct categories, including P-, P+, P++, P+++, Adjacent, and Dirty.

To facilitate comprehensive analysis, each WSI image was segmented into patches of 1024*1024 pixels. Within each patch, we performed segmentation and classification utilizing the SOLOv2 model. SOLOv2 was a simple, direct, fast and powerful instance segmentation model, which significantly improved the detection speed and produced better results through matrix non-maximum suppression technology. The outcomes were consolidated, and a detailed statistical analysis was carried out to quantify the total area and proportion occupied by distinct categories, including P-, P+, P++, P+++, Adjacent, and Dirty. These categories were essential in characterizing the staining intensity and extent of G3BP1 expression within the tissue. Finally, based on these detailed findings, a score was assigned to each individual case providing a quantitative representation of the G3BP1 status. The entire process is illustrated in Fig 1, elucidating the systematic steps involved in our model’s application and the subsequent scoring of G3BP1 in each case. This methodology ensures a thorough and standardized evaluation, contributing to the accuracy and reliability of the deep learning-based analysis.

#### 2.4.1 Data processing

The dataset was meticulously annotated using Labelme software by Yuting Zhan, a pathology doctor, to delineate the contours and categories of targets within WSI images. The annotated targets included P-, P+, P++, P+++, Adjacent, and Dirty, encompassing a comprehensive spectrum of staining categories. Given the substantial size of WSI images, segmentation was conducted with overlapping cuts, employing a step size of 256 pixels. This segmentation resulted in images sized at 1024*1024 pixels facilitating more manageable processing.

Subsequently, the segmented data was organized into a COCO dataset to facilitate effective model training. Data augmentation techniques including random flipping, random resizing, and data normalization, were applied to enhance the robustness and generalizability of the model. The dataset then partitioned into a training set, a validation set and a test set, maintaining a balanced ratio of 6:2:2. The painstaking process of curating and augmenting this dataset guarantees that the model can proficiently capture the subtleties of G3BP1 staining patterns, which also enhances the model’s dependability and precision in following analyses.

#### 2.4.2 Segmentation model & training

**Fig 2:**
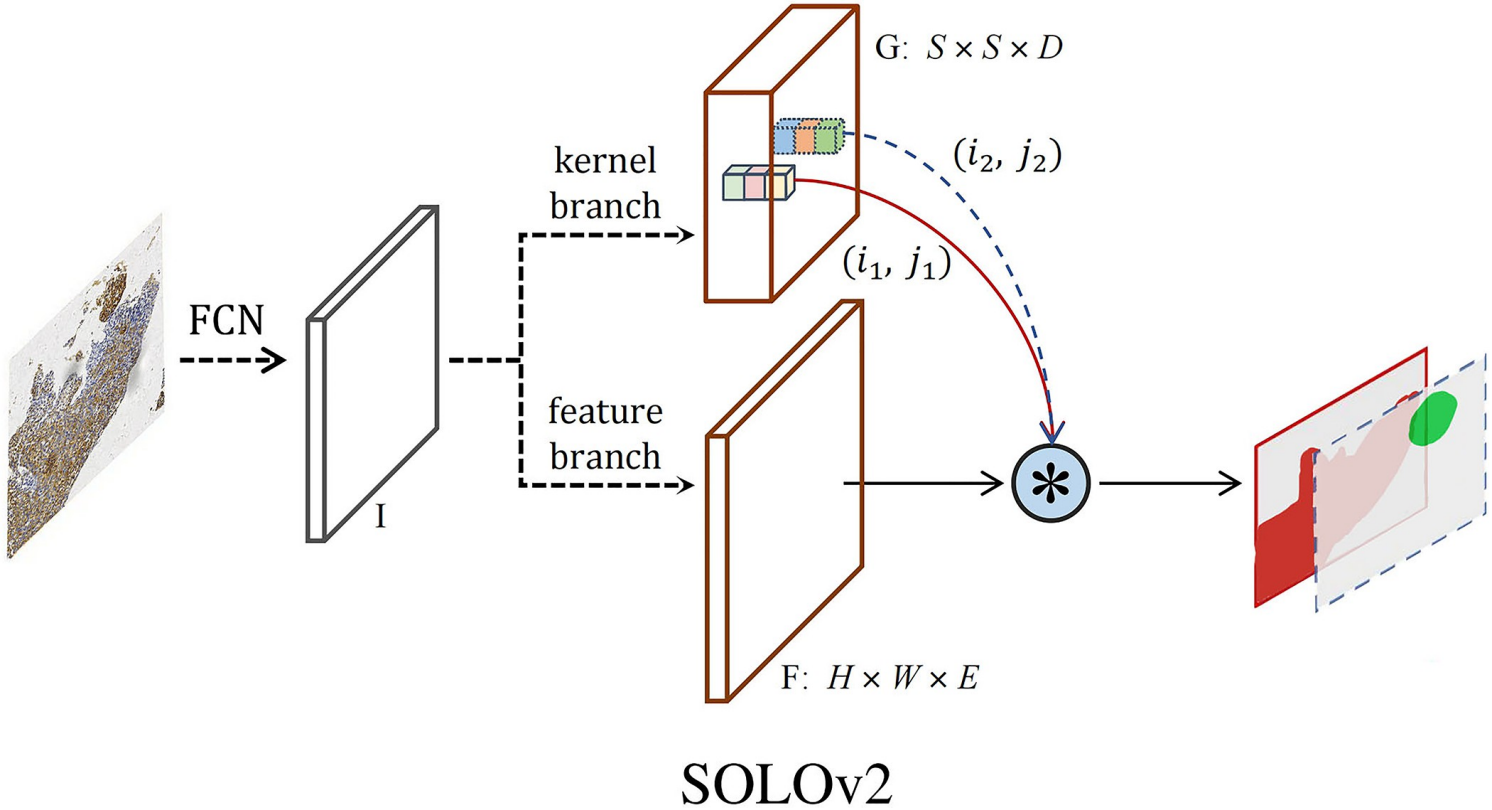
The SOLOv2 instance segmentation model. The image underwent an initial input into the Fully Convolutional Networks (FCN) to generate Feature Map I. This feature map underwent traversal through two distinct pathways: the Kernel Branch and the Feature Branch. The Kernel Branch executed convolution on the feature map, yielding Mask Kernel G, while the Feature Branch performed convolution on the same map, resulting in Mask Feature F. Subsequently, G was p subdivided into S*S grids. Whenever an object’s center fell within a grid cell, the convolution kernel corresponding to that cell, along with the Mask Feature F, were utilized for dynamic convolution computation. This process generated the object’s mask and category.

For segmentation purposes, we employed the SOLOv2 instance segmentation model, with the overall structure depicted in in Fig 2. The image underwent an initial input into the Fully Convolutional Networks (FCN) to generate Feature Map I. This feature map underwent traversal through two distinct pathways: the Kernel Branch and the Feature Branch. The Kernel Branch executed convolution on the feature map, yielding Mask Kernel G, while the Feature Branch performed convolution on the same map, resulting in Mask Feature F. Subsequently, G was p subdivided into S*S grids. Whenever an object’s center fell within a grid cell, the convolution kernel corresponding to that cell, along with the Mask Feature F, were utilized for dynamic convolution computation. This process generated the object’s mask and category [17]. This intricate segmentation approach, inherent to the SOLOv2 model, enables precise delineation and categorization of objects within the images, ensuring the accuracy of subsequent analyses. The comprehensive structure of the model facilitates robust training, vital for its application in scoring G3BP1 staining patterns.

For the model training phase, we employed Stochastic Gradient Descent (SGD) as the optimizer, with an initial learning rate set at 0.01 and a momentum value of 0.9. The training process spanned 50 epochs, utilizing Dice Loss as the mask loss function and Focal Loss as the classification loss function. These choices were made to ensure an effective balance between precise mask delineation and accurate category classification. To evaluate the model’s performance and generalizability, we utilized the mean Average Precision (mAP) value on the validation set. This metric acts as a thorough gauge of the model’s precision regarding mask creation and category forecasting, offering important understandings into its total effectiveness and preparedness for further examinations.

#### 2.4.3 Statistical score

It was computed the total area for each of the six categories within the case: P-, P+, P++, P+++, Adjacent, and Dirty. Subsequently, we determined the percentage of each category’s area in relation to the total area. To calculate the total score, it was established a base score for positive categories: (P+) = 1, (P++) = 2, and (P+++) = 3, reflecting their respective levels of positivity. The final score for the case was calculated as follows: Case Final Score = percent area (P+) * 1 + percent area (P++) * 2 + percent area (P+++) *3.

## 3. Results

### 3.1 Analysis of segmentation model training process

The dataset encompassed 275 IHC stained WSIs representing the G3BP1 protein. These WSIs were systematically split into three subsets: the training set, the validation set, and the test set, maintaining a distribution ratio of 6:2:2. Within the domain of deep learning, our primary focus was on the meticulous fine-tuning of model parameters to minimize the discrepancy between predicted and actual outcomes within input data. The evaluation metric employed for assessing this disparity was the loss rate. This metric served as a critical benchmark, quantifying the disparity between model predictions and actual results. A lower loss rate was indicative of a more closely aligned correlation between the model’s predictions and actual outcomes, thus signifying superior performance. The meticulous analysis of the segmentation model training process provides valuable insights into the model’s optimization and readiness for subsequent analyses.

**Fig 3:**
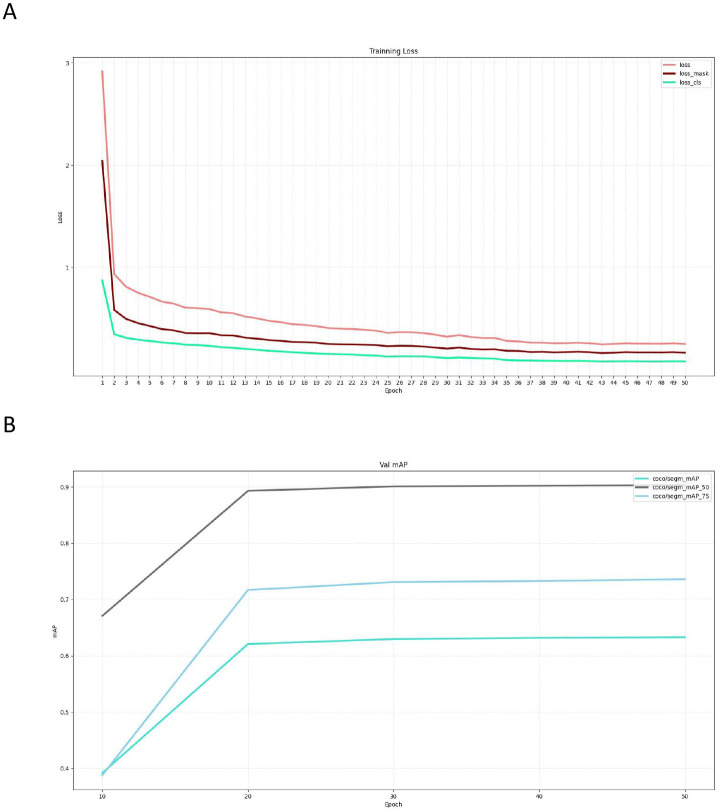
The training and the assessment of the G3BP1 molecular marker scoring model. (A) It was revealed a rapid decline in loss within the initial 2 epochs, signaling the model’s swift adaptation to the specified task. As the training epochs advanced, the model consistently demonstrated a convergence between predicted outcomes and actual results. (B) Across the initial 20 epochs, there was a progressive increase in mAP. During the post the 30th epoch, the mAP value stabilized. Training was concluded at the 50th epoch.

Leveraging our extensive dataset, we undertook the training the SOLOv2 segmentation model. Throughout this process, we diligently documented the incurred loss. The graphical representation of these outcomes, as illustrated in Fig 3A, revealed a rapid decline in loss within the initial 2 epochs, signaling the model’s swift adaptation to the specified task. As the training epochs advanced, the model consistently demonstrated a convergence between predicted outcomes and actual results. This observation highlights the model’s adeptness for the assigned task and underscores its robust performance on the training data. The detailed analysis of the loss trends provides crucial insights into the model’s learning dynamics and its capability to effectively capture the nuances within the G3BP1 staining patterns.

### 3.2 Assessment of the G3BP1 molecular marker scoring model

Simultaneously, to evaluate the model’s efficacy on the test set, we employed mAP, mAP50, and mAP75 as evaluation metrics on the validation set. Widely utilized for assessing object detection algorithms, these metrics measured the average accuracy under varying Intersection over Union (IoU) thresholds. Specifically, mAP50 and mAP75 represented the average accuracy calculated at IoU (Intersection over Union) thresholds of 0.5 and 0.75, respectively. Higher values of mAP, mAP50, and mAP75 indicated improved accuracy in model detection. Model assessments on the validation set were conducted at intervals of 10 epoch intervals during the training phase, with mAP, mAP50, and mAP75 values recorded, as illustrated in Fig 3B. Across the initial 20 epochs, there was a progressive increase in mAP, indicating continuous improvement in model performance throughout the learning phase. Post the 30th epoch, the mAP value stabilized, suggesting convergence towards an optimal state. The training came to an end in the 50th epoch, indicating the attainment of a strong and high—performing G3BP1 molecular marker scoring model.

### 3.3 Impact of expression of G3BP1 on the overall survival of patients with NPC

**Fig 4:**
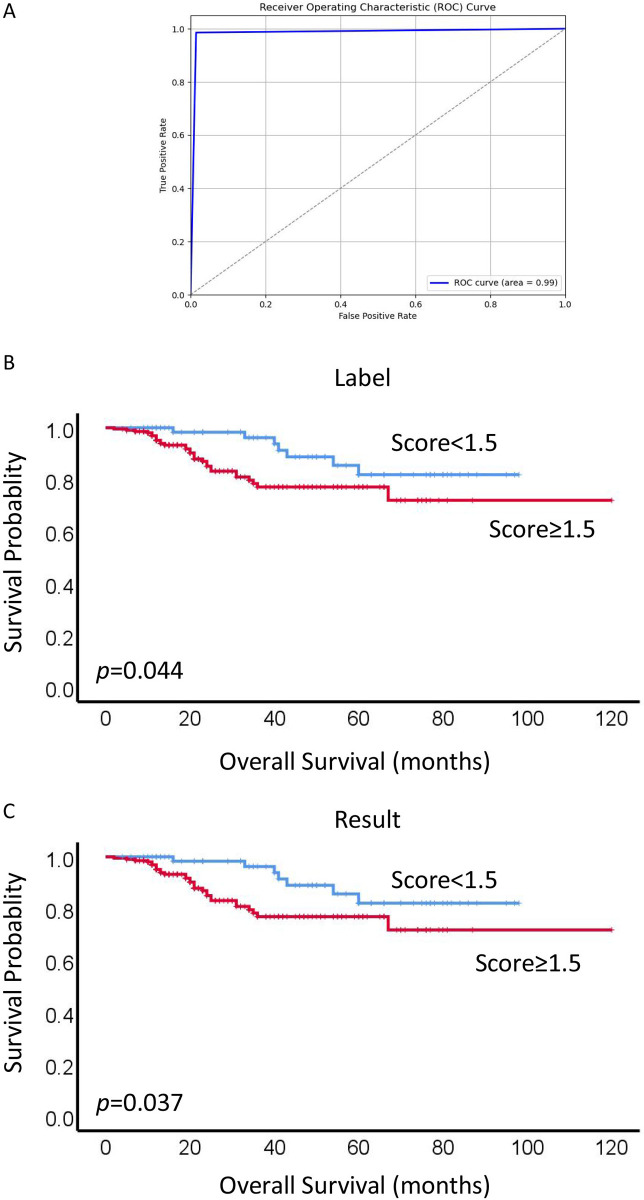
Impact of expression of G3BP1 on the overall survival of patients with NPC. (A)The ROC curve was drew to evaluate the model and threshold value, and the cut-off number was reasonable for 1.5. (B and C)The statistical significance of both label group and result group was determined using the log-rank test respectively.

The evaluation was based on the intensity and extent of staining, and a semi-quantitative evaluation for G3BP1 was labeled by researcher, following being calculated by the established model. Staining intensity for G3BP1 was appraised as 0 (P-), 1 (P+), 2 (P++), and 3 (P+++). Staining extent was recorded and calculated by the percent of actual area. The staining intensity and extent were both based on tumor cells rather than lymphocytes. The final score for the case was calculated as follows: Case Final Score = percent area(P+) * 1 + percent area(P++) * 2 + percent area(P+++) *3. Two sets of data were available and showed in S1 Table. The Full score was 3, and therefore, staining scores <1.5 and ≥1.5 were regarded as low expression and high expression for G3BP1, respectively. We drew the ROC curve to evaluate the model and threshold value. The area under the curve is 0.99, indicating that the accuracy of the model is very good and the selection of cut-off value was reasonable (depicted in Fig 4A).

To comprehensively assess the model implications and investigate the influence of G3BP1 expression on the overall survival rates (OS) of NPC patients, Kaplan-Meier analysis was applied to plot survival curves. The statistical significance of both label group and result group was determined using the log-rank test respectively (depicted in Fig 4B and 4C). Univariate survival analysis, conducted via the log-rank test, revealed significantly lower OS rates for NPC patients exhibiting high expression of G3BP1 proteins in both label group and result group (*p* = 0.044 and *p* = 0.037, label group and result group, respectively. Furthermore, to explore whether high expression of G3BP1 proteins served as an independent prognostic factor for NPC patients, multivariate Cox proportional hazard regression analysis was conducted (refer to Tables 2 and 3). In multivariate analysis encompassing various patients features such as gender, age, histological type, LNM status, clinical stages and G3BP1 expression, high G3BP1 expression emerged as an independent poorer prognostic factors for NPC patients (*P* = 0.038 and *P* = 0.029, label group and result group, respectively). These findings underscore the potential of G3BP1 as a critical biomarker for prognostic assessments in NPC patients.

**Table 2:** Summary of multivariate of Cox proportional regression for overall survival in 275 cases of NPC.

Parameter	SE	Wald	Sig.	Exp (B)	95.0% CI for Exp (B)	
					Lower	Upper
**Gender**	0.456	3.944	0.047	0.404	0.165	0.988
**Age**	0.482	1.417	0.234	0.563	0.219	1.449
**Histological type**	0.620	1.418	0.234	0.478	0.142	1.611
**LNM status**	1.025	6.860	0.009**	0.068	0.009	0.509
**Clinical stages**	0.409	0.467	0.494	0.756	0.339	1.686
**G3BP1 (Label)**	0.425	4.303	0.038*	2.415	1.050	5.556

Abbreviations: CI, confidence interval; Exp (β), odds ratio; LNM, lymph node metastasis; Radio, radiotherapy; Chemo, chemotherapy

Note: multivariate analysis of Cox regression

*P<0.05

** *P*<0.01.

**Table 3:** Summary of multivariate of Cox proportional regression for overall survival in 275 cases of NPC.

Parameter	SE	Wald	Sig.	Exp (B)	95.0% CI for Exp (B)	
					Lower	Upper
**Gender**	0.458	3.730	0.053	0.413	0.169	1.013
**Age**	0.482	1.252	0.263	0.583	0.226	1.500
**Histological type**	0.620	1.373	0.241	0.483	0.143	1.631
**LNM status**	1.024	7.083	0.008**	0.066	0.009	0.488
**Clinical stages**	0.410	0.423	0.515	0.766	0.343	1.710
**G3BP1 (result)**	0.425	4.762	0.029*	0.395	0.172	0.910

Abbreviations: CI, confidence interval; Exp (β), odds ratio; LNM, lymph node metastasis; Radio, radiotherapy; Chemo, chemotherapy

Note: multivariate analysis of Cox regression

*P<0.05

** *P*<0.01.

## 4. Discussion

Artificial intelligence (AI) has emerged as a revolutionary power within pathology and has made substantial contributions to the field of personalized medicine. Its applications span clinically applied AI, aimed at relieving, and more explorative research AI pathologists of repetitive and quantitative routine tasks, and more explorative research AI, which involves predict protein expression, forecasting patients prognosis, and anticipating the response to treatments[10, 14, 19–21]. Computational pathology, a subset of AI, holds immense potential in both pathological diagnosis and scientific research. In clinical settings, computational pathology addresses the repetitive and subjective aspects of pathology work, enhancing efficiency and accuracy [15, 19]. For example, it has shown promise in thin-prep cytologic test (TCT), peritoneal lavage cytology, and tumor classification, significantly reducing the workload of pathologists and improving diagnostic precision [22–24]. The advent of computer-assisted diagnosis is considered a disruptive technology that could replace routine tasks performed by pathologists.

The development towards precision medicine has expanded the role of pathologists beyond diagnosis. Computational pathology, fueled by big data, can offer detailed information such as molecular classification, drug sensitivity, and patient prognosis. Studies have demonstrated the potential of Hematoxylin & Eosin (H&E) staining in predicting molecular classification, paving the way for a broader application of this technique in predicting various protein expressions [10, 14, 25]. This forecast can expand to other proteins that need quantification such as Ki-67, a good marker of proliferation [26–29]. Computational pathology has also explored the combination of multiple IHC markers (such as CD8, CD20, CD68 and Ki-67) to explore the immune microenvironment, leading to the establishment of new molecular classification that can guide clinical strategies [30]. Moreover, scientists are seeking the possibility using the combination of diagnosis-specific IHC staining to replace H&E staining diagnosis and obtain the creditable results [31].

This project focused on the IHC staining of G3BP1, a protein studied by the research group over the years [4, 5]. The evaluation of G3BP1 is known to be labor-intensive and subjective, prompting the adoption of computational pathology. The project’s framework demonstrates impressive performance in evaluating G3BP1 staining and predicting the prognosis of NPC patients. The study is consistent with previous research, confirming the oncogenic role of G3BP1 and highlighting its association with the poor prognosis of NPC patients.

Nevertheless, there are acknowledged limitations in the project. The universality of the framework needs to be testing, particularly its application to other types of cancer, assessing precision, specificity and potential optimization. Furthermore, optimizing the framework to incorporate additional clinical information could enhance the model’s accuracy, aligning more closely with the principles of precision medicine and providing more nuanced guidance for clinical treatment and maximizing patient benefit. Continued research and further refinement will contribute to the broader adoption and effectiveness of computational pathology in clinical settings.

## Supporting information

S1 Table Naso sample area percent result. (XLSX)

## References

[1] Luo W. Nasopharyngeal carcinoma ecology theory: cancer as multidimensional spatiotemporal "unity of ecology and evolution" pathological ecosystem. Theranostics. 2023. 13(5): 1607–1631. doi: 10.7150/thno.82690 37056571 PMC10086202

[2] Wong K, Hui EP, Lo KW, et al. Nasopharyngeal carcinoma: an evolving paradigm. Nat Rev Clin Oncol. 2021. 18(11): 679–695. doi: 10.1038/s41571-021-00524-x 34194007

[3] Xia C, Dong X, Li H, et al. Cancer statistics in China and United States, 2022: profiles, trends, and determinants. Chin Med J (Engl). 2022. 135(5): 584–590. doi: 10.1097/CM9.0000000000002108 35143424 PMC8920425

[4] Zhan Y, Wang W, Wang H, et al. G3BP1 Interact with JAK2 mRNA to Promote the Malignant Progression of Nasopharyngeal Carcinoma via Activating JAK2/STAT3 Signaling Pathway. Int J Biol Sci. 2024. 20(1): 94–112. doi: 10.7150/ijbs.85341 38164170 PMC10750281

[5] Zheng H, Zhan Y, Zhang Y, et al. Elevated expression of G3BP1 associates with YB1 and p-AKT and predicts poor prognosis in nonsmall cell lung cancer patients after surgical resection. Cancer Med. 2019. 8(16): 6894–6903. doi: 10.1002/cam4.2579 31560169 PMC6853815

[6] Zhan Y, Chen X, Zheng H, et al. YB1 associates with oncogenetic roles and poor prognosis in nasopharyngeal carcinoma. Sci Rep. 2022. 12(1): 3699. doi: 10.1038/s41598-022-07636-z 35260638 PMC8904596

[7] Chen L, Xie G, Feng J, et al. Overexpression of FADD and Bcl-XS proteins as novel prognostic biomarkers for surgically resected non-small cell lung cancer. Cancer Biomark. 2021. 30(2): 145–154. doi: 10.3233/CBM-190018 33104018 PMC12499981

[8] Xu Y, Quan Z, Zhan Y, et al. SSTR2 positively associates with EGFR and predicts poor prognosis in nasopharyngeal carcinoma. J Clin Pathol. 2023. 10.1136/jcp-2023-208987 PMC11671960 37758305

[9] Williams C, Seligmann JF, Elliott F, et al. Artificial Intelligence-Assisted Amphiregulin and Epiregulin IHC Predicts Panitumumab Benefit in RAS Wild-Type Metastatic Colorectal Cancer. Clin Cancer Res. 2021. 27(12): 3422–3431. doi: 10.1158/1078-0432.CCR-21-0120 33888518

[10] Shamai G, Livne A, Polónia A, et al. Deep learning-based image analysis predicts PD-L1 status from H&E-stained histopathology images in breast cancer. Nat Commun. 2022. 13(1): 6753. 36347854 10.1038/s41467-022-34275-9 PMC9643479

[11] Lemaître G, Martí R, Freixenet J, Vilanova JC, Walker PM, Meriaudeau F. Computer-Aided Detection and diagnosis for prostate cancer based on mono and multi-parametric MRI: a review. Comput Biol Med. 2015. 60: 8–31. doi: 10.1016/j.compbiomed.2015.02.009 25747341

[12] Jiang Y, Yang M, Wang S, Li X, Sun Y. Emerging role of deep learning-based artificial intelligence in tumor pathology. Cancer Commun (Lond). 2020. 40(4): 154–166. doi: 10.1002/cac2.12012 32277744 PMC7170661

[13] Deng S, Zhang X, Yan W, et al. Deep learning in digital pathology image analysis: a survey. Front Med. 2020. 14(4): 470–487. doi: 10.1007/s11684-020-0782-9 32728875

[14] Farahmand S, Fernandez AI, Ahmed FS, et al. Deep learning trained on hematoxylin and eosin tumor region of Interest predicts HER2 status and trastuzumab treatment response in HER2+ breast cancer. Mod Pathol. 2022. 35(1): 44–51. doi: 10.1038/s41379-021-00911-w 34493825 PMC10221954

[15] Han Z, Lan J, Wang T, et al. A Deep Learning Quantification Algorithm for HER2 Scoring of Gastric Cancer. Front Neurosci. 2022. 16: 877229. doi: 10.3389/fnins.2022.877229 35706692 PMC9190202

[16] Wang CW, Lin KY, Lin YJ, Khalil MA, Chu KL, Chao TK. A Soft Label Deep Learning to Assist Breast Cancer Target Therapy and Thyroid Cancer Diagnosis. Cancers (Basel). 2022. 14(21). doi: 10.3390/cancers14215312 36358732 PMC9657740

[17] Wang X, Zhang R, Kong T, Li L, Shen C. SOLOv2: Dynamic and Fast Instance Segmentation.

[18] Zhan Y, Feng J, Lu J, Xu L, Wang W, Fan S. Expression of LEF1 and TCF1 (TCF7) proteins associates with clinical progression of nasopharyngeal carcinoma. J Clin Pathol. 2019. 72(6): 425–430. doi: 10.1136/jclinpath-2019-205698 30918012

[19] Schüffler P, Steiger K, Weichert W. How to use AI in pathology. Genes Chromosomes Cancer. 2023. 62(9): 564–567. doi: 10.1002/gcc.23178 37254901

[20] Viswanathan VS, Toro P, Corredor G, Mukhopadhyay S, Madabhushi A. The state of the art for artificial intelligence in lung digital pathology. J Pathol. 2022. 257(4): 413–429. doi: 10.1002/path.5966 35579955 PMC9254900

[21] Al Zorgani MM, Ugail H, Pors K, Dauda AM. Deep Transfer Learning-Based Approach for Glucose Transporter-1 (GLUT1) Expression Assessment. J Digit Imaging. 2023. 36(6): 2367–2381. doi: 10.1007/s10278-023-00859-0 37670181 PMC10584776

[22] Zhou M, Zhang L, Du X, et al. Hierarchical pathology screening for cervical abnormality. Comput Med Imaging Graph. 2021. 89: 101892. doi: 10.1016/j.compmedimag.2021.101892 33744789

[23] Chen X WZ, He Y HZ, Wang Q ZK, Zhou W WP, Shan F LZ, Ji. Accurate and Rapid Detection of Peritoneal Metastasis from Gastric Cancer by AI‐Assisted Stimulated Raman Molecular Cytology. Adv Sci (Weinh). 2023. 10(21). doi: 10.1002/advs.202300961 37114845 PMC10375130

[24] Zhu L SH, Wei H WC, Shi S ZF, Yan R LY, He T WL,. An accurate prediction of the origin for bone metastatic cancer using deep. EBioMedicine. 2023 Jan;87:104426. 36577348 10.1016/j.ebiom.2022.104426 PMC9803701

[25] Tewary S, Mukhopadhyay S. HER2 Molecular Marker Scoring Using Transfer Learning and Decision Level Fusion. J Digit Imaging. 2021. 34(3): 667–677. doi: 10.1007/s10278-021-00442-5 33742331 PMC8329150

[26] Liu Y, Li X, Zheng A, et al. Predict Ki-67 Positive Cells in H&E-Stained Images Using Deep Learning Independently From IHC-Stained Images. Front Mol Biosci. 2020. 7: 183. 32903653 10.3389/fmolb.2020.00183 PMC7438787

[27] Vesterinen T, Säilä J, Blom S, Pennanen M, Leijon H, Arola J. Automated assessment of Ki-67 proliferation index in neuroendocrine tumors by deep learning. APMIS. 2022. 130(1): 11–20. doi: 10.1111/apm.13190 34741788 PMC9299468

[28] Fulawka L, Blaszczyk J, Tabakov M, Halon A. Assessment of Ki-67 proliferation index with deep learning in DCIS (ductal carcinoma in situ). Sci Rep. 2022. 12(1): 3166. doi: 10.1038/s41598-022-06555-3 35210450 PMC8873444

[29] Negahbani F, Sabzi R, Pakniyat Jahromi B, et al. PathoNet introduced as a deep neural network backend for evaluation of Ki-67 and tumor-infiltrating lymphocytes in breast cancer. Sci Rep. 2021. 11(1): 8489. doi: 10.1038/s41598-021-86912-w 33875676 PMC8055887

[30] Meier A, Nekolla K, Hewitt LC, et al. Hypothesis-free deep survival learning applied to the tumour microenvironment in gastric cancer. J Pathol Clin Res. 2020. 6(4): 273–282. doi: 10.1002/cjp2.170 32592447 PMC7578283

[31] Salvi M, Manini C, López JI, Fenoglio D, Molinari F. Deep learning approach for accurate prostate cancer identification and stratification using combined immunostaining of cytokeratin, p63, and racemase. Comput Med Imaging Graph. 2023. 109: 102288. doi: 10.1016/j.compmedimag.2023.102288 37633031

